# On Hemorrhagic Diseases of the New-Born, with Report of a Case Due to Duodenal Ulcer

**Published:** 1913-09

**Authors:** W. J. H. Pinniger

**Affiliations:** Surgical Registrar, Bristol General Hospital.


### ON HEMORRHAGIC DISEASES OF THE NEW-BORN, WITH REPORT OF A CASE DUE TO DUODENAL ULCER.

BY

W. J. H. PINNIGER, M.D., B.S. Lond., M.R.C.S., L.R.C.P.,
*Surgical Registrar, Bristol General Hospital.*

---

HEMORRHAGES occurring in new-born infants are grouped together under one title, "The Hemorrhagic Diseases of the New-Born." These have been subdivided into four classes, as follows :—[1]

1. Syphilis hemorrhagica neonatorum.
2. Morbus maculosus neonatorum.
3. Bleeding from ulcers of œsophagus, stomach or intestine.
4. Epidemic hæmoglobinuria (Winckel's disease).

To take the last first—epidemic hæmoglobinuria. This consists of jaundice, fever, gastro-intestinal symptoms, with albumin and methæmoglobin in the urine. This symptom-complex is not liable to confusion with other forms of the hemorrhagic disease, but requires to be diagnosed from simple icterus neonatorum, in which there may be blood in the urine. This disease is probably not a separate entity, but merely one expression of the general disease, for *post-mortem* swelling of the spleen and punctiform hemorrhages in the internal organs have been found.

With regard to No. 1—syphilis hemorrhagica neonatorum—there would appear to be no reason for assigning a special class to these cases. They seem to be simply instances of an association in the same individual of the two diseases, syphilis and the hemorrhagic disease. There is no reason why a syphilitic child should be exempt from the disease, but rather the reverse, its degenerated condition rendering it more liable to the onset and continuance of hemorrhages ; but, on the other hand, in so few of the recorded cases of hemorrhagic disease, is there any evidence of syphilis in the parents or child, that the occurrence of this disease, in the absence of any definite luetic signs, is no reason for suspecting its subject to be syphilitic.

The position of the third class—that in which a definite lesion of the intestinal tract is found—is more uncertain. The details of the recorded cases are in many instances very meagre ; but since in one recorded *post-mortem* there were other lesions found—petechiæ on the cheek and small infarcts in the lung—it is likely that this class also belongs to the same general disease, and at any rate, inasmuch as the local condition is undiagnosable during life, so far as treatment is concerned it necessarily comes in with the others.

We come, then, to consider the hemorrhagic disease of the new-born as a whole.

The onset is within the first week. The main manifestations of the disease are—

Hemorrhages from mouth, nose, stomach, bowels, umbilicus, severally or in any combination.

Cutaneous and submucous petechiæ or extravasations.

Jaundice in some cases.

Rise of temperature in some cases.

The course is short. Many cases are fatal, death occurring within a week, and being due either to anæmia from the losses of blood or to the child being brought to such a condition of weakness from these losses that it does not take its food, and dies from inanition, though no further bleeding may occur. If recovery takes place the bleedings gradually cease, and there is no liability to recurrence in later life.

*Frequency.*—Townsend [2] in 1891 reported 45 cases in a series of 6,700 deliveries. Shukowsky [3] in 1907 reported 29 cases in a series of 30,000 deliveries. Other writers give the frequency at about 1 in 1,000. One would suppose the difference is dependent on what cases the writers consider grave enough to merit inclusion.

*Mortality.*—Townsend in 1891 reported a mortality of 79 per cent. in 609 collected cases, and 78 per cent. in 32 of his own. In 1894 he reported a mortality of 62 per cent. in 50 cases of his own. Schöppler [4] gives the mortality as 56 per cent.

*Pathological Anatomy.*—On *post-mortem* examination various lesions are found. Amongst these are—

Extravasations of various size into subcutaneous or submucous tissues.[1, 5, 6, 7.]

Extravasations of various size into internal organs, *e.g.* liver, spleen, lungs.

Patches of extravasation in the mucous membrane of the intestine, with round-celled exudation, but without loss of substance. [4]

Hemorrhagic erosion of mucous membrane of stomach and intestine.

Rupture of a large vein in the stomach. [8]

Gastric ulcer. [9]

Duodenal ulcer.[1, 9, 10.]

Finally, in many cases nothing abnormal is discovered *post-mortem.*[1 6 10]

*Cause.*—Many suggestions have been made as to the cause of this condition, the most important being these :—

Difficult labour with mechanical injuries to the internal organs.

Syphilis.

Causes connected with the umbilical cord: (1) Infection. (2) Connected with ligature of the cord, *e.g.* embolism, or congestion in portal or hepatic circulation.

Hæmophilia.

Non-hæmophilic diminution in coagulability of the blood.

(i) The first of these suggested causes—mechanical injuries—cannot be the cause of prolonged generalised bleedings, and the number of cases in which it gives rise to hemorrhage at all is very small. In most recorded cases of hemorrhage of the new-born the labour has been perfectly natural and normal.

(ii) The association of syphilis with this disease has already been mentioned, and its infrequency as a cause of it pointed out.

(iii) Infection via the cord. Most infantile maladies have been put down to infection through the cord, and seeing how large the umbilical vessels are, and how directly they are connected with important organs, any infection of the clot in them is very potent for ill; but in the cases of this disease reported in detail a history of any septic condition of the cord is conspicuous by its absence, so that this cannot be a frequent cause.

Causes associated with ligature of the cord:—

(*a*) Embolism. The separation of emboli from the thrombus in the umbilical vein was thought by Lister[6] to explain the duodenal ulcer found in his case and the small infarcts of the lung. This cause could only be presumed to be present, however, in cases with definite local lesions, and these are relatively very few in number.

(*b*) Congestion in the portal circulation. According to Schöppler,[4] ligature of the cord causes acute hyperæmia of the stomach and intestine, and in conditions of defective resistance this hyperæmia, together with the further irritation induced by food, leads to inflammation and fatal hemorrhage. This, however, can only apply to cases with gastro-intestinal hemorrhage alone.

(iv) Hæmophilia. These cases, though they resemble hæmophilics in many ways, are not instances of that disease, for the following reasons :—

(*a*) The affection is purely a temporary phenomenon. If the patient's strength holds out and the amount of blood lost at any one time is not too great, the disease will come to an end, and this favourable termination occurs in about half the cases.

(*b*) Females are affected as often as males.

(*c*) There is no history of "bleeders" in most of the cases.

(*d*) In those cases which recover there is no tendency to recurrence of the bleeding.

(*e*) Hæmophilia is very rare within the first year of life. According to Larrabee, there are only thirty-seven cases in the literature.

(v) Diminution in the coagulbility of the blood. The actual evidence for this theory is very small in amount.

Schwartz and Ottenberg[11] found that the coagulation time in one case was delayed four days. Mosenthal,[5] in reporting a case cured by transfusion, considers the bleeding was due to diminished coagulability, though no observation on the coagulation time is recorded. Bigelow,[7] reporting three cases cured by injection of fresh serum, comes to the same conclusion, though again without experimental demonstration of the diminution, the cause in his opinion being lack of fibrin ferment.

A very great deal more investigation is needed before it can be said in which or in what combination of these conditions, if in any, lies the cause of these bleedings. If one may hazard a guess it seems likely that the diminution in the coagulability will prove to be the underlying cause ; but even if this be so, there must be some other exciting cause, possibly amongst those that have been mentioned, to start the bleeding in any one place.

*Treatment.*—This, as the death rate shows, is unsatisfactory, though there is reason for hope that newer methods may improve matters. The disease is a self-limited one, that is to say, that the bleeding tends to stop of itself, but very many patients die in the meantime of loss of blood.

The greatest amount of success has been met with along one of two lines, viz. : (1) Direct transfusion of human blood ; (2) Subcutaneous injection of fresh rabbit's serum.

Treatment on these lines is based on the results of Weil's experiments. He showed that bleeding in hæmophilic conditions is due not to the presence of an anti-coagulation ferment, but to the absence or modification of certain substances normally present in the blood. If to some normal fresh blood some hæmophilic serum is added, no change in the coagulation time of the normal blood is produced ; but if to some fresh hæmophilic blood a small quantity of normal fresh serum is added, the coagulation time of the hæmophilic blood is brought down to normal. The inference was that the same thing might be brought about in the living body by the introduction of normal human blood directly into the circulation or by the subcutaneous injection of some fresh serum.

In the first method there is the additional advantage that good normal blood is poured into the patient's impoverished circulation, so that the child starts life afresh, as it were, with a new stock of normal blood. The drawback to the method is its difficulty, transfusion by means of a cannula having been found impossible, and end-to-end anastomosis of the radial artery of the donor with the femoral vein of the child must be resorted to.

At least six cases of this procedure are recorded, all within the last four years.[5, 11, 12, 13, 14.] Of these three were successful cures and one improved for a time, the result in the other two not being clearly stated. In the four mentioned the improvement was sudden and striking, a child which was moribund at the commencement of the operation being at the end a strong, lusty infant. Bleedings diminished from the first and soon ceased. The one which relapsed was the case in which the coagulation time is known. Before operation it was delayed four days ; improvement was marked, and lasted some days.

Symptoms began then to reappear and the coagulation time also lengthened. Ultimately the child died.

In the second method—subcutaneous infusion of serum—5 c.c. of fresh rabbit's serum were introduced subcutaneously, and the results obtained were again most striking. Three cases are detailed by Bigelow [7] in his paper describing the treatment, and all three recovered, the bleeding rapidly ceasing after but one, or in one case two, injections of the serum.

Other subsidiary methods of treatment which have been tried or recommended are:—

Injection of gelatin[9] (15 c.c. of a 2 per cent. solution being boiled for six hours, then cooled to 100° F., and then injected. Holtschmidt reported recoveries in five consecutive cases treated by this method).

Internal administration of suprarenal gland.

Local application of adrenalin, where the bleeding spot is accessible.

In cases where the bleeding is from the intestinal tract, castor oil to relieve congestion and cold alum whey as a food if artificial feeding is being used, the feeds being small and frequent if the child is breast-fed.

With regard to duodenal ulcer in the new-born, a very complete account of the cases hitherto published is given in one of the chapters in the recent edition of Moynihan's book on *Duodenal Ulcer*. There are only sixteen cases recorded in which death occurred within a week of birth, and only six more with a fatal termination within the first two months. The most generally-accepted theory as to the causation is that of thrombosis of the umbilical vein with scattering of portions of the thrombus into the general circulation and infarction of the duodenum, digestion of the infarct producing the ulcer.

Another case, not included in Moynihan's list, very similar to the one reported below, is described by Fabre and Rhenter in the *Bulletin de la Société d'Obstétrique de Paris* for 1912.

The history of the present case is as follows: The child, a female, an eight months' child, was born on November 8th, 1911. It was well-nourished and cried strongly, but was obviously premature, the skin being red and still covered with lanugo, and the nails not reaching to the ends of the fingers. On the second day of its life a curious stool was noticed, but it was not reported. On the third day three stools were passed, and all of these contained blood. The first contained a considerable quantity of bright red blood, the second a small quantity of bright red blood, and the third was entirely composed of tarry material. On the fourth day three more black tarry stools were passed, and in the evening an injection of 5 c.c. of serum was given. Normal serum was not obtainable, so diphtheria antitoxin was used instead. In spite of the bleeding, the child was still looking fairly well, cried strongly, and took the breast well. On the fifth day another black motion having been passed in the night, a second similar injection of the same serum was given. Two more black stools were passed that day and two more early on the sixth day, but the third on the sixth day was nearly normal—yellow with just a little dark material in it. The child was now failing very badly. It was too weak to suck. The milk was withdrawn from the breast by the pump and the child spoon-fed with it, a few drops of brandy being added to each feed. On the seventh day two normal stools were passed, and there was no bleeding. The breast milk had given out, and the child was being fed with a few drops of modified milk every hour with two drops of brandy in each feed. It was very weak. On the eighth day the motions were again normal, but the child was getting progressively weaker. On the ninth day it vomited about half an ounce of blood and passed a very large black stool, and died a few hours later, not, however, in a blanched condition.

Thus in the nine days of its life the child passed twelve blood-containing stools and vomited blood once. There was no hemorrhage from any other source. The cord was quite normal and healthy.

With regard to its antenatal history, the mother was a primipara, healthy, and there was no reason to suspect syphilis in the father. The labour was natural and easy, the cord being tied at the usual time, viz. as soon as the pulsation had ceased.

A partial *post-mortem* was all that the circumstances permitted, which is much to be regretted. This consisted in removal of the stomach and intestines for later more careful examination, and inspection of the rest of the abdominal organs. The abdominal cavity was normal ; the stomach was distended ; the appearance of the intestines normal. On examining the interior of the alimentary tract a few congested patches were found in the stomach and small intestine, and in the first part of the duodenum, half an inch from the pylorus, a small ulcer was discovered. This was elliptical in shape, with its long axis in the long axis of the gut, and measured 5 mm. by 3 mm. It had a slightly raised edge which shelved away to normal mucous membrane all round, and towards the base internally. The ulcer was shallow, but on holding it up to the light its base was semi-transparent, and apparently consisted only of peritoneum. The peritoneal coat was normal. The small intestine contained a good deal of red turbid fluid, like thin anchovy sauce, but no other lesion than this was found in it nor in the large bowel. The other abdominal organs were apparently normal.

REFERENCES.

[1] Osler, *Principles and Practice of Medicine*, 1910, p. 755.
[2] Townsend, *Boston M. and S. J.*, 1891, cxxv. 218.
[3] Shukowsky, *Arch. f. Kinderh.*, 1907, xlv. 321.
[4] Schöppler, *Centralbl. f. allg. Path.*, 1910.
[5] Mosenthal, *J. Am. M. Ass.*, 1910, liv. 1613.
[6] Lister, *Tr. Path. Soc. Lond.*, 1899, l. 111.
[7] Bigelow, *J. Am. M. Ass.*, 1910, lv. 400.
[8] Soltau Fenwick, *Disorders of Digestion in Infancy and Childhood*, 1897, p. 312.
[9] Goodhart and Still, *Diseases of Children*, 1905.
[10] Moynihan, *Duodenal Ulcer*, 1912.
[11] Schwartz and Ottenberg, *Am. J. M. Sc.*, 1910, cxl. 17.
[12] Lambert, *Med. Rec.*, 1908, lxxiii. 885.
[13] Swain, Jackson, Murphy, *Boston M. and S. J.*, 1909, clxi. 407.
[14] Lespinasse and Fisher, *Surg., Gynec. and Obst.*, 1911, xii. 40.

